# Physiological Traits for Shortening Crop Duration and Improving Productivity of Greengram (*Vigna radiata* L. Wilczek) Under High Temperature

**DOI:** 10.3389/fpls.2019.01508

**Published:** 2019-12-04

**Authors:** Partha Sarathi Basu, Aditya Pratap, Sanjeev Gupta, Kusum Sharma, Rakhi Tomar, Narendra Pratap Singh

**Affiliations:** ^1^Division of Basic Science, ICAR - Indian Institute of Pulses Research, Kanpur, India; ^2^Division of Crop Improvement, ICAR - Indian Institute of Pulses Research, Kanpur, India

**Keywords:** thermo-tolerance, acquired thermotolerance, chlorophyll fluorescence, photosynthesis, sucrose synthase

## Abstract

Greengram is an important protein-rich food legume crop. During the reproductive stage, high temperatures cause flower drop, induce male sterility, impair anthesis, and shortens the grain-filling period. Initially, 116 genotypes were evaluated for 3 years in two locations, and based on flowering, biomass, and yield attributes, they were grouped into four major clusters. A panel of 17 contrasting genotypes was selected for their heat tolerance in high-temperature greenhouses. The seedlings of the selected genotypes were exposed to heat shock in the range 37°C–52°C and their recovery after heat shock was assessed at 30°C. The seedlings of EC 398889 turned completely green and rejuvenated, while those of LGG 460 failed to recover, therefore, EC 398889 and LGG 460 were identified as heat-tolerant and heat-sensitive genotypes, respectively. Except for EC 398889, the remaining genotypes could not survive after heat shock. Fresh seeds of EC 398889 and LGG 460 were planted in field and pollen fertility and sucrose-synthase (SuSy) activity in grains were assessed at high temperatures. The pollen germination and SuSy activity were normal even at temperatures beyond 40°C in EC 398889 and high SuSy activity enabled faster grain filling than in LGG 460. The precise phenotyping demonstrated significant differences in the light-temperature response of photosynthesis, chlorophyll fluorescence imaging of quantum yield (Fv/Fm), and electron transport rate (ETR) between heat-tolerant (EC 398889) and heat-sensitive (LGG 460) genotypes. Molecular profiling of selected accessions showed polymorphism with 11 SSR markers and the markers CEDG147, CEDG247, and CEDG044 distinguished tolerant and sensitive groups of accessions.

## Introduction

Greengram (*Vigna radiata* L. Wilczek), also known as mungbean, is an important grain legume containing a high amount of digestible protein, amino acids, sugar, minerals, soluble dietary fibres, and vitamins. It is cultivated across seasons, in different environments, and in variable soil conditions in the South and South-East Asia, Africa, South America, and Australia (Parihar et al., 2017). The productivity and adaptability of greengram are adversely affected by several abiotic stresses including heat, drought, salinity, and water-logging, which affect crop growth and development by altering physiological processes and the plant-water relationship (Dreesen et al., 2012; Bita and Gerats, 2013; Suzuki et al., 2014; Kaur et al., 2015; Zandalinas et al., 2017; Landi et al., 2017). Several studies have reported a reduction in growth and development of legumes because of high-temperature stress (Tzudir et al., 2014; Hanumantha Rao et al., 2016). Greengram thrives most effectively at temperatures between 30°C and 40°C, however, significant flower shedding occurs at temperatures beyond 40°C (Zinn et al., 2010; Sita et al., 2017). Rainey and Griffiths (2005) reported that the abscission of reproductive organs is the primary determinant of yield under heat stress in several grain legumes. The production is considerably influenced by changes in the photoperiod and temperature across the growing regions of greengram extending from low to high latitudes. Because greengram is a quantitative short-day plant (Chauhan and Williams, 2018), short day length at low latitude hastens flower initiation, and the plants rapidly reach the reproductive phase without adequate vegetative biomass production. By contrast, long photoperiod at high latitudes delays the onset of the reproductive phase, but the biomass is adequate and has a high leaf area index.

Crops grown at high latitudes are often exposed to high temperatures beyond the threshold tolerance limit (40°C). The interactive effects of photoperiod and temperature in greengram are inadequately understood although both factors are crucial determinants of grain yield; therefore, photo-thermo insensitivity is a major attribute in breeding strategy in the development of greengram varieties with higher stability across diverse climatic conditions. Generally, a higher mean temperature hastens flowering, or a lower mean temperature delays flowering in all photoperiods (Sharma and Dhanda, 2014; Sharma et al., 2016). Singh and Singh (2011) and Lateef et al. (2018) reported temperature × flowering interactions in greengram with high mean temperatures (24°C to 28°C) and long photoperiods (15 to 16 h). Grain yield reduction in heat stress in several plant species has been reported to be associated with a decrease in photosynthetic capacity because of altered membrane stability (Savchenko et al., 2002; Salvucci and Crafts-Brandner, 2004; Zhang et al., 2009; Zhang and Sharkey, 2009; Egorova et al., 2011; Kumar et al., 2011; Horváth et al., 2012; Bita and Gerats, 2013; Kumar et al., 2013; Rakavi and Sritharan, 2019) and enhanced maintenance respiration (Reynolds et al., 2007) along with a reduction in radiation-use efficiency. However, photosynthesis is the most sensitive physiological process impaired by heat stress (Crafts-Brandner and Salvucci, 2002; Marchand et al., 2005; Kepova et al., 2005; Yang et al, 2006; Wang et al., 2009).

Decrease in photosynthesis at high temperatures could result from structural and functional disruptions of chloroplasts, reduction of chlorophyll, inactivation of chloroplast enzymes (Dekov et al., 2000; Langjun et al., 2006) or both stomatal and nonstomatal limitation (Wahid et al, 2007). Oxidative stress can cause lipid peroxidation and consequently membrane injury, protein degradation, and enzyme inactivation (Meriga et al., 2004).

High temperatures adversely affect starch and sucrose synthesis through a reduction in the activity of sucrose phosphate synthase and ADP-glucose pyrophosphorylase (Rodriguez et al., 2005; Zhao, 2013). Several reports have indicated that reproductive failure in heat stress could possibly be due to impaired sucrose metabolism in the leaves, developing grains, and anthers as well as the inhibition of sucrose transporters which reduces the availability of triose phosphates to the developing pollen grains and causes reproductive failure (Kaushal et al., 2013; Kaushal et al., 2016). Crops exposed to high temperature are often subjected to oxidative stress-producing reactive oxygen species (ROS), which are highly toxic to cellular functions in plants because they damage nucleic acids and cause protein oxidation and lipid peroxidation; this oxidative damage eventually causes cell death (Suzuki et al., 2012; Tuteja et al., 2012). ROS toxicity during various stresses is considered to be one of the major causes of low crop productivity worldwide (Vadez et al., 2012). An increase in the activity of antioxidant enzymes, such as guaiacol peroxidase (GPX) and catalase (CAT), plays a significant role in minimizing the toxic effects of stress-induced ROS production (Anderson and Sonali, 2004; Hassan and Mansoor, 2014). The present investigation is an attempt at large-scale screening of germplasm across diverse climates, identifying lines with photo-thermo insensitivity, thermotolerance, and, high grain-filling rates, as well as at deciphering the mechanisms of heat tolerance in lines with high production potential.

## Materials and Methods

### Field Trial

A set of 116 greengram genotypes, including exotic lines and cultivars, were grown during summer in augmented design during April–May for 3 consecutive years from 2015 to 2017 at two contrasting growing regions at different latitudes, namely, Vamban, Tamil Nadu, India (10.20°N, 78.50° E; day length 11:30 to 12:45 h) and Kanpur (26.4° N; 80.3° E; day length 12:30 to 14:0 h) in an augmented design with four checks, namely, Samrat, IPM 99-125, IPM 02-3, and IPM 02-14, which were replicated at an interval of 20 test genotypes. Recommended package of practices were followed for successful crop growth. The performance of genotypes and their grouping was assessed on the basis of phenology, biomass, pod fill duration, harvest index, and grain yield for pooled data of adjusted mean of each trait over 3 years for cluster analysis. The selected 17 high-yielding and stable genotypes were sown in three replications under natural field conditions at Kanpur for detailed study, whereas for precision phenotyping, the plants were grown in a controlled environmental chamber (High Point, Taiwan). The naturally-lit greenhouse experiment conducted using 17 high-yielding and 11 low-yielding greengram genotypes led to the identification of two degrees of thermotolerance when the plants were grown under 45°C and 25°C maximum and/minimum temperature, respectively, and 14 h day length. For all the traits for phenotyping, replicated samples (3–5) were used and significant levels of treatment means were worked out using factorial design of analysis of variance test. These promising genotypes, namely, EC 398889 with a high pod bearing capacity and LGG 460 without pods, were selected for further studies in detail to decipher their differential sensitivity towards high temperature and ability to set pods at high temperature.

### Physiological Characterization of Contrast Genotypes for Heat Tolerance

The physiological characterization of selected two contrasting photoinsensitive genotypes with high and low yields was conducted for assessing their sensitivity to heat stress using different parameters, namely, membrane stability, acquired thermotolerance (ATT), chlorophyll index, chlorophyll fluorescence, pollen germination, sucrose synthase activity for sink strength, and protein and molecular profiling.

### Membrane Stability Test

The fully expanded young leaves of the photoinsensitive, EC 398889 and LGG 460 genotypes grown in a naturally-lit greenhouse were sampled for membrane stability test. In the test, electrolyte leakage was assessed after treatment using a conductivity meter model Hanna (USA). This treatment was repeated in a session for 1 h at 40°C (C 1) followed by 100°C (C2) and the electrical conductivity of this solution at the two temperature was measured separately. The relative membrane stability index was calculated using the formula given by Blum and Ebercon (1981), as Membrane Stability or injury index = C1/C2, where C1 = Electrical conductivity (EC µS) at test temperature 40°C for 1h; and C2 = Electrical conductivity (EC µS) at 100°C for 1 h.

### Chlorophyll Estimates

For instant chlorophyll estimation in the plants grown at a high temperature, a noninvasive technique was used to assess the chlorophyll status or “greenness index,” which used MinoltaModel 502 Soil Pant Analytical Development (SPAD).

### Acquired Thermotolerance

Acquired thermotolerance (ATT) of the seedlings of the heat-sensitive (LGG 460) and heat-tolerant (EC 398889) greengram genotypes was assessed by the methods described by Porter et al. (1994). The germinating seedlings were subjected to temperature shock treatment starting from 37°C to 52°C with an increment of 2°C and 2 h of incubation at each temperature. After reaching the peak temperature (52°C) the treatments were reversed to 37°C in the descending order. Cell viability test was assessed using the 2,3,5 Triphenyl tetrazolium chloride (TTC) reduction assay on the seedlings after treatment at normal (37°C) and high (52°C) temperatures. After heat shock for 2 h, the cells were cooled down to room temperature and 1% TTC solution was added to the cells followed by overnight incubation. A purple color developed because of the formation of formazan in the tissues that remained viable and could restore respiration. The level of (ATT) was determined by measuring the percentage reduction of TTC to formazan using the following formula: ATT (%) = (OD_37°c-52°c_/OD _37°c_) ×100.

### Specific Leaf Area

A young leaf disc of 1-cm diameter from 10 plants each of 17 high-yielding and 11 low-yielding selected field-grown greengram genotypes was excised during the podding stage when average maximum and minimum temperature reached approximately 40°C/30°C. The leaf discs were dried and weighed ten leaf discs of each genotype. The specific leaf area (SLA) was calculated by the total area of ten leaf discs over their total dry weight and expressed as cm^2^g^−1^. The values of SLA were regressed with SPAD Chlorophyll Meter Reading (SCMR) values of same leaf and obtained the relationship.

### Carbon Isotope Discrimination

At 50 days after sowing, replicated samples of fully turgid green leaves of 17 high- yielding and 11 low-yielding field-grown genotypes adjacent to podding cluster was excised (pooled samples). The leaves of each genotype were dried gradually at temperatures less than 80°C using a hot air oven for 3 days and were fine-powdered in a mill. Carbon isotope composition was determined on 1 mg sample with Isotopic Ratio Mass Spectrometer (Thermo Finnigan, Bremen, Germany) at the University of Agricultural Sciences, Bangalore, India. Carbon isotope discrimination (D) was calculated according to Farquhar et al. (1989). The values of delta carbon regressed with SLA values of the same leaf to obtain the relationship.

### Fluorescence Image Analysis

Field-grown replicated leaf samples collected from well-irrigated plants of EC 398889 and LGG 460 were used for chlorophyll fluorescence studies at the flowering stage. Following chlorophyll fluorescence, studies were immediately conducted using a fluorescence imaging system (Mess & Regeltechnik, Waltz, Germany). Initially, uniform specific geometrical areas of a single leaf of each genotype were selected to obtain ETR. The dark-adapted leaves were used before getting the light curve and initial fluorescence values, Fo and Fm, were used for further calculation using the following formula:

ETR=Quantum yield × PAR × 0.5 × absorptivitry

while

$$\begin{array}{c} \text{ETR}=\text{Photosynthetic}\;\text{electron}\;\text{transport}\;\text{rate};\;\text{PAR}=\\ \;\;\text{Photosynthetic}\;\text{active}\;\text{radiation} \end{array}$$

The absorptive parameter describes the fraction of incident light, which is absorbed. The factor 0.5 considers that only half of the absorbed quanta is distributed to PS II (under steady state conditions), light curve of individual selection was obtained by increasing the order of irradiance till ETR became light saturated.

### Gaseous Exchange

The plants of EC 398889 and LGG 460 were raised in two chambers maintained at maximum/minimum temperature regimes of 40°C/30°C and 25°C/18°C with a 14-h light period at 450 µmol photon m^−2^s^−1^ in a controlled growth chamber (Hi- Point, Taiwan). The plants grown at 25°C/18°C were considered to be grown at a low temperature (LT), while those grown at 40°C/30°C were considered to be grown at a high temperature (HT). The photosynthesis and other gaseous exchange parameters of the LT- and HT-grown plants were measured using a portable photosynthesis system (Model Li-COR 6400 xt, USA) under saturating light intensity 1500 µmol photon m^−2^s^−1^.

### Pollen Germination Test

The pollen germination test was conducted on field-grown greengram genotypes EC 398889 and LGG 460 by exposing excised flowers of these genotypes placed over moistened filter paper to different controlled temperature regimes (29°C, 32°C, 35°C, 37°C, 39°C, 41°C, and 43°C) for 2 h for acclimatization. The germinating pollen tubes were stained using 10% acetocarmine solution. Germination of fresh pollen grains was assessed using the sucrose-hanging-drop culture. A drop of germination medium (15% aqueous sucrose solution containing 200 mg H_3_BO_3_, 100 mg Ca (NO_3_)_2_, 100 mg MgSO_4_, 100 mg KNO_3_, and 50 mg EDTA) was placed on a coverslip and pollen dusted onto the drop. The coverslip was then inverted and placed over a concave depression on a slide, using glycerol to seal the coverslip and prevent desiccation. Then incubated for 24 hours at 29°C, 32°C, 35°C, 37°C, 39°C, 41°C, and 43°C. Following this, pollens were stained using acetocarmine solution and viewed under microscope (Leica DM 2000, Germany).

### Sucrose Synthase Activity

Freshly developed pods of the field-grown genotypes EC 398889 and LGG 460 were excised when they attained a length of approximately 1 cm. Sucrose synthase activities were determined in developing grains at various stages by using the method described by Wang et al. (1993) with slight modifications. Tissue samples (approximately 2 g of fresh tissue) were cut into small pieces and homogenised in extraction buffer. To assay enzyme activity, an aliquot of the extract was desalted using a microcentrifuge desalting procedure using Sephadex G-25 columns. The solution thus obtained was incubated for 15 min at 30°C with 20 µM of fructose and 20 µM of UDP-glucose in 90 µL of 50 mM HEPES buffer (pH 8.5) containing 15 mM MgCl_2_, and the reaction was stopped by adding 120 µL of 1 N NaOH. The amount of enzyme solution and reaction time were previously determined to be in the linear range of the reaction. The sucrose synthase activity was assayed in the forward direction only.

### Protein Profiling

Seedlings were allowed to grow for 3 weeks under controlled conditions in a plant growth chamber with an illumination of 460 µmol photons m^−2^s^−1^and 14-h day length. Both the genotypes (EC 398889 and LGG 460) were grown in two temperature regimes, namely, 30°C/20°C and 43°C/35°C. A standard protocol was followed. Accordingly, the treated leaf tissue (0.5 g) was homogenized in buffer for protein extraction. The protein concentration in the supernatants of the samples were estimated following the method described by Peterson (1977). A 12.5% separating gel containing 375 mM Tris-HCl, pH 8.8, 0.1% (w/v) SDS, 0.05% (w/v) ammonium persulfate and 0.4 µL ml^−1^ TEMED was used for resolving the polypeptides. Protein markers containing polypeptides of different molecular weight were run along with protein samples extracted from the test samples. Approximately 15–30 µg of protein sample was loaded in each well.

### Molecular Profiling

Total genomic DNA was isolated according to the method of Doyle and Doyle (1987) with slight modifications (Gupta et al., 2013). The quantity as well as quality of extracted DNA were checked by comparison with 300 ng of standard ƛDNA. The working DNA sample was diluted to a standard concentration of 25 ng/µL. The DNA samples used for molecular marker analysis by using 79 SSR primer pairs derived from adzuki bean (Wang et al., 2004) were screened to detect polymorphism among groups of heat-tolerant, moderately tolerant, and sensitive greengram lines. Polymerase chain reaction was carried out following standard procedure. PCR products obtained were resolved by electrophoresis on 3% agarose gel for 3 h in 19 TAE buffer, stained with ethidium bromide and photographed using a Gel Documentation System (Uvitech, Cambridge, UK). Polymorphic markers were distinguished on the basis of the presence or absence of amplified product and difference in allele size by comparison with 100-bp DNA ladder.

## Statistical Analysis

The observations made on field evaluation were subjected to statistical analysis of augmented design as described by Fedrer (1961). The analysis takes into account the variability among blocks measured by standard check varieities, according to which the values of entries were subjected to comparison. Significance of treatment mean difference or least square difference was estimated using multivariate factorial analysis variance (ANOVA) alongwith standard error of means and deviation. The genotypes were grouped into different clusters based on Ward’s method using squared Euclidian distances by using the statistical software SPSS version 16.0 (SPSS, Chicago, USA) program.

## Results

**Figure 1:**
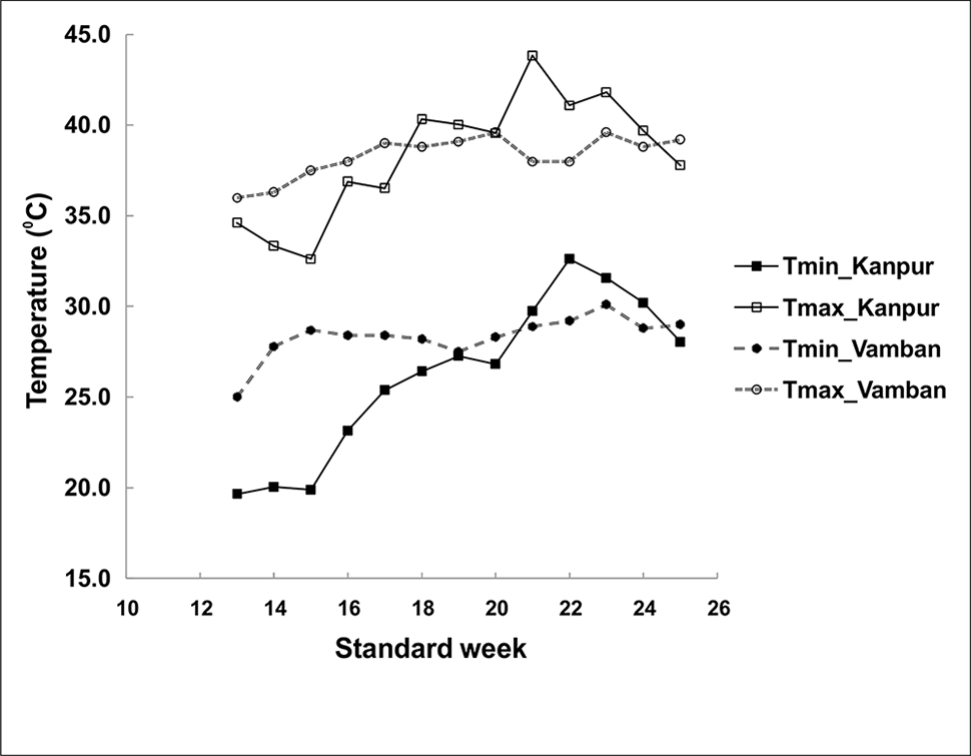
Temperature regime during crop duration of greengram at two experimental sites.

**Figure 2:**
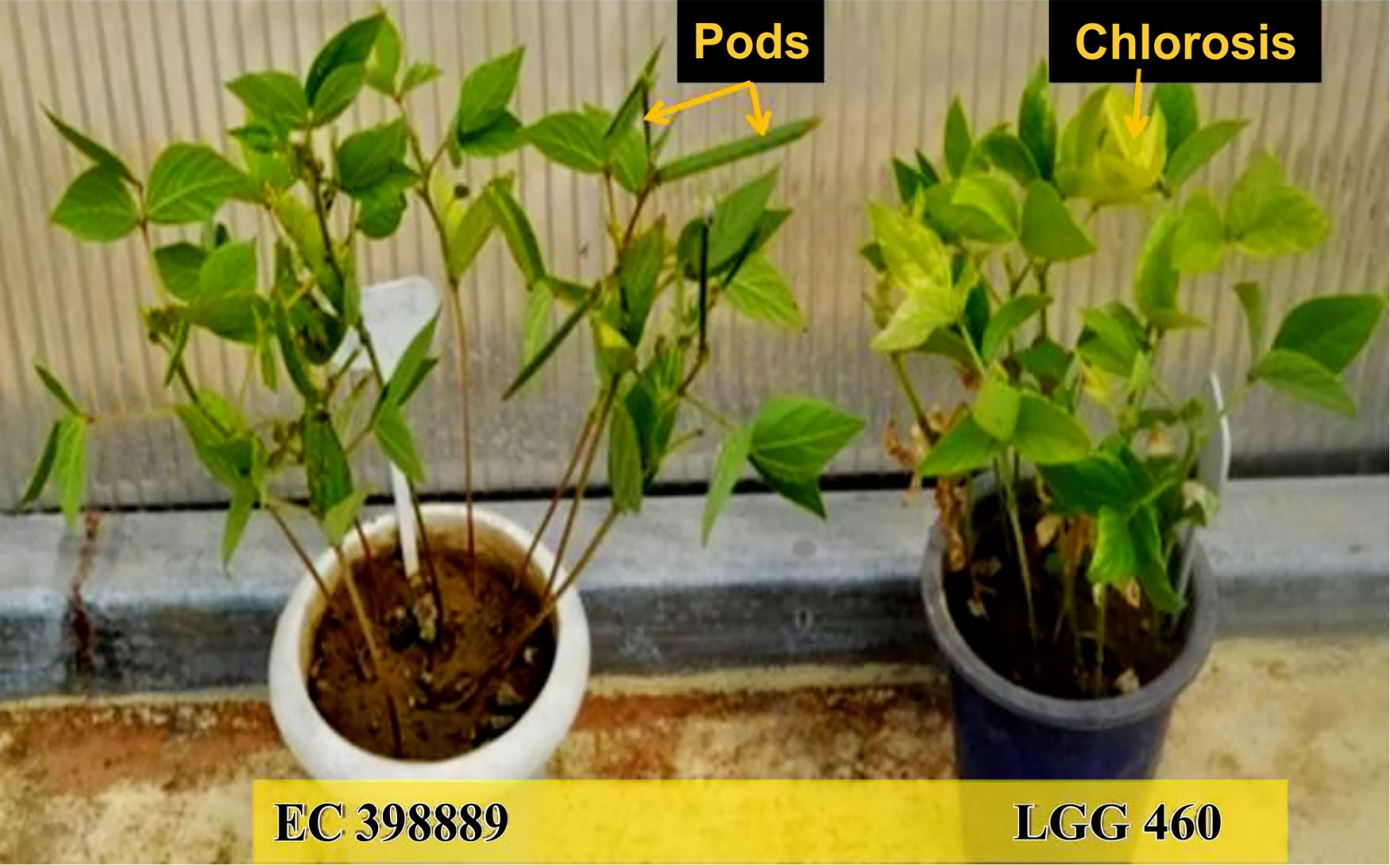
Performance of a heat tolerant (EC 398889) and heat sensitive (LGG 460) genotypes at high temperature (45/25^0^C max/min) and 14 h day length (Heat tolerant genotype showed pod formation while sensitive genotype without pods at high temperature).

**Figure 3:**
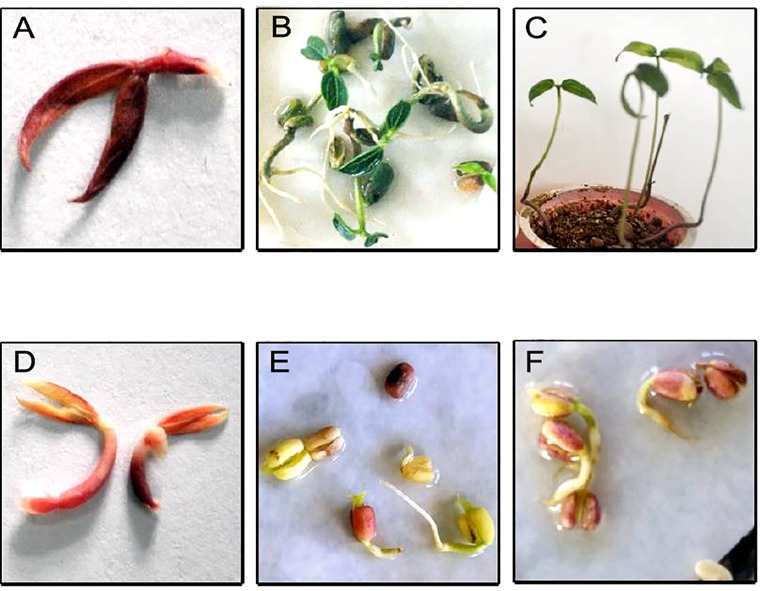
Seedling viability and regeneration after heat shock at 52^0^C in heat tolerant (EC 398889) **(A**–**C)** and heat sensitive (LGG 460) genotype **(D**–**F)**. **(A** and **D)** showing results of TTC test; **(B** and **E)** showing amount of chlorophyll accumulation and **(C** and **F)** showing rejuvenation or failure of normal growth of plants after heat treatment.

To identify the greengram genotype with adequate thermotolerance, the criteria were chosen as yield potential and stability across different locations and temperature regimes (**Figure 1**). The test population of greengram at Vamban and Kanpur classified into four major clusters and 11 subsclusters were distinctly different from each other in the phenological and yield attributing traits (**Table 1**). Three years of experimentation resulted in the identification of contrasting green- gram genotypes with stable high and low yields (**Table 2**). All the 116 genotypes showed earlier flowering (25–32 days) and pod setting, lower mean biomass (700–3600 kg/ha), and lower grain yield (343–1745 kg/ha) at Vamban than at Kanpur (**Table 2**). The days to first flower, biomass and harvest index differed considerably in most of the test genotypes in Vamban and Kanpur (**Table 2**). Out of 116 genotypes, 17 accessions had high and stable yields, while 11 had stable low yields across two locations and 3 years of experiments, although genotype × location × years interaction was highly significant at the 1% level (**Table 2**). Therefore, investigating heat tolerance of 17 high and 11 low-yielding greengram genotypes under controlled grenhouse conditions at a HT regime (max/min 45°C/25°C) throughout the entire period of crop growth became necessary. This greenhouse experiment led to identification of two contrasting genotypes, namely LGG 460 (heat-sensitive genotype) and EC 3398889 (heat-tolerant genotype) (**Figure 2**). Based on the ability to set pods at HT in EC 398889, it was tentatively designated a heat-tolerant genotype, while LGG 460, which failed to form pods, was assumed to be a heat-sensitive genotype. The membrane stability index was moderately high in EC 398889 as compared with LGG 460, however chlorophyll index as assessed by SCMR (SPAD chlorophyll meter reading) remained higher in EC 398889 at HT regime (**Table 2**). ATT was considerably higher in EC 398889 (76.8%) than in LGG 460 (34.5%). This phenomenon was further validated by TTC test as indicated by the differences in intensity of purple formazan formation when TTC solutions was added to the seedlings of EC 398889 and LGG 460 after heat shock treatment treated at 52°C for 2 h (**Figures 3A, D**). Stepwise heat shock treatment from 37°C to 52°C with increments of 2°C at each step and its reversal to normal temperature provided crucial evidence regarding adaptation of EC 398889 towards HT because this genotype showed TTC positive staining, greening, and normal restoration of plant growth after severe heat shock (**Figures 3B, C**), while LGG 460 completely lost seed viability after HT shock (**Figures 3E, F**).

**Table 1:** Clustering of greengram genotypes based on yield attributing traits across two locations and over three years of trials.

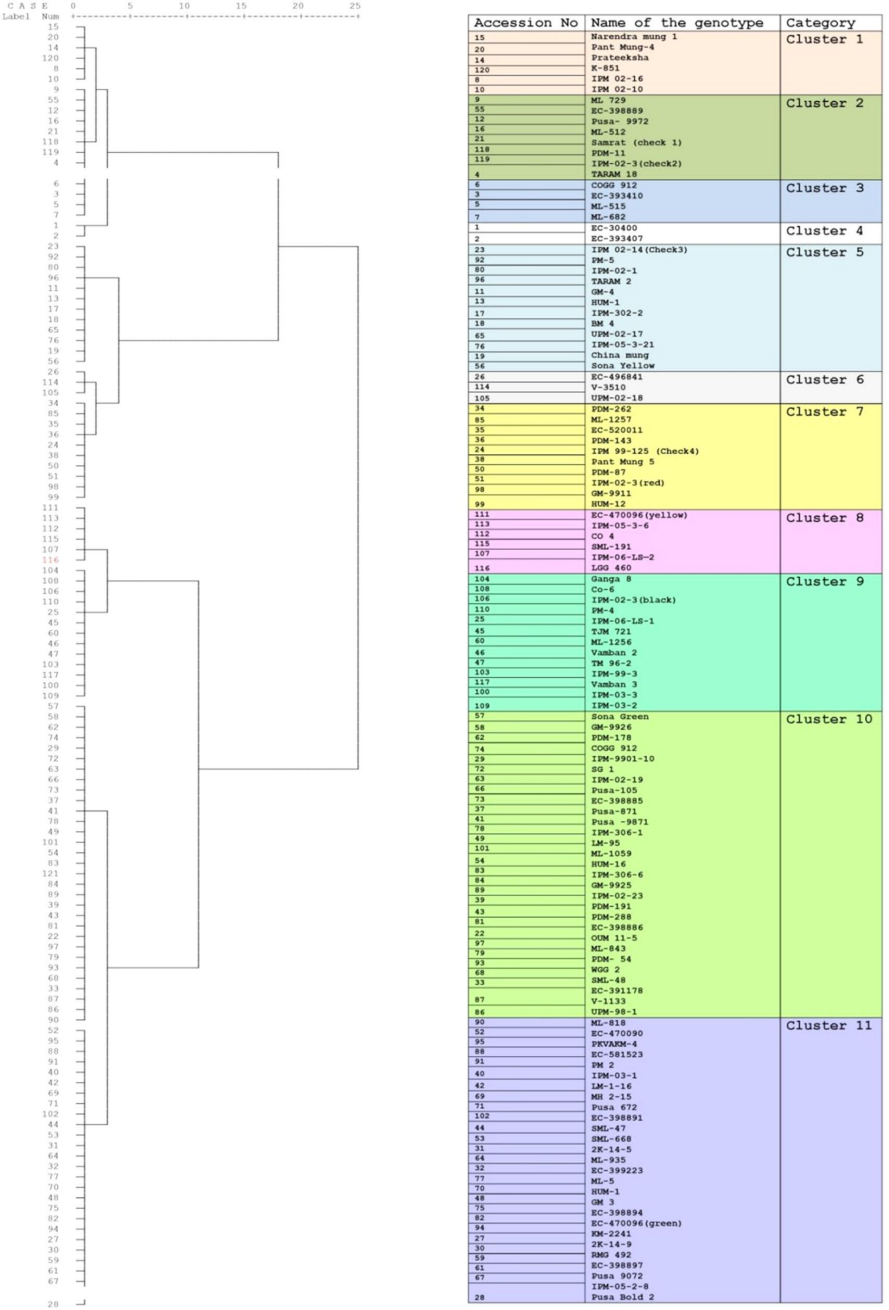

**Table 2:** Categorization of greengram genotypes into Group 1, 2, and 3 based on yield performance across two locations and over 3 years of trials.

Group 1	Group 2	Group 3
Genotypes with stable high yield	Genotypes with unstable Yield	Genotypes with stable low yield
Stable yield across locations & years (High grain yield at both experimental stations)	Variation in grain yield (high at Kanpur while low at Vamban) across locations and years. List of genotypes below having < 1000 Kg/ha yield at Vamban but > 1500 kg/ha at kanpur	Low yielding genotypes at both the locations ≤500kg /ha
Ganga 8,HUM-12,,China mung 1, IPM 02-14, IPM 99-125, PDM-262, EC-520011, **EC 398889**, PDM-87, IPM-02-3(red), Sona Yellow, HUM-16, UPM-02-17, IPM-02-1, TARAM 2,GM-9911, ML-1256	EC-393407, TARAM 18, COGG 912, ML 729,, Pusa- 9972, ML 512, Samrat, Pusa Bold 2, IPM-9901-10, RMG 492, 2K-14-5, EC-399223, SML-48, Pusa-871, Pant Mung 5, PDM-191, IPM-03-1, Pusa -9871,LM-1-16, PDM-288, SML-47, TJM 721, Vamban 2, TM 96-2, GM 3, EC-470090, SML-668, EC-398897, Pusa 9072,, IPM-02-19, ML-935, Pusa-105, IPM-05-2-8, SML-48, HUM-1, SG 1, EC-398885, COGG 912, EC-398894, IPM-306-1, PDM- 54, EC-398886, EC-470096 (green), IPM-306-6, UPM-98-1, V-1133, ML-818, PM 2, PM-5, WGG 2, PKVAKM-4, ML-843, IPM-03-3, ML-1059, EC-398891, IPM-99-3, IPM-06-LS—2, Co-6, IPM-02-3(black), IPM-03-2, PM-4, EC- 470096 (yellow), IPM-05-3-6,, PDM-11, K-851,Sona green	MH 2-15, Pusa 672, ML-5,EC-581523, NSB 007,CO4 KM-2241, SML-191 Kopergaon, **LGG 460**, Vamban 3
**Range of phonological and yield attributing traits of same panel of genotypes across two locations**		
Grain yield (Kg/ha)	**Kanpur**	**Vamban**
	642-3094	343-1745
Days to First flower	35-39	25-32
Biomass (kg/ha)	700-3800	700-3600
Pod fill duration (days)	8-15	15-20
Harvest index (%)	7-34	17-59

Interaction of Genotype x Location x Year : Significant at ≤0.01.

Bolded text represented the greengram genotype (EC 398889) with consistent stable high yield while genotype (LGG 460) having consistent low stable yield performance at two locations and over three years of trial conducted.

**Figure 4:**
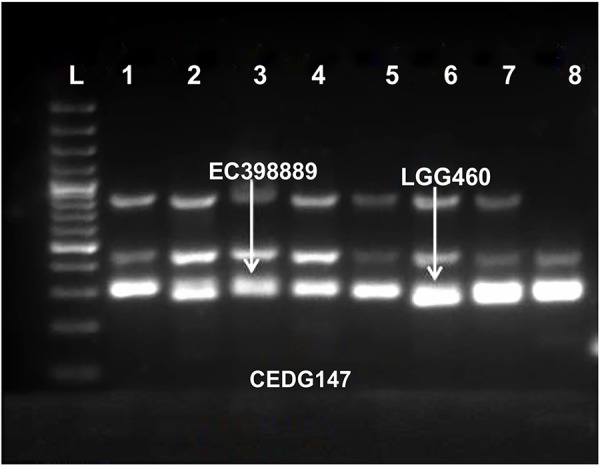
Molecular profiling of amplified DNA of leaf samples extracted from selected heat sensitive and heat tolerant greengram genotypes. Marker CEDG 147, L-100 bp ladder; 1. (HUM 12); 2. (Ganga 8); 3. (EC 398889); 4. (IPM-02-03); 5.(IPM-02-14); 6. (LGG 460); 7. (Kopergaon); 8. (NSB 007).

Molecular profiling of identified genotypes was conducted using 79 SSR markers of which 11 were found to be polymorphic. These markers exhibited considerable genetic variability among different genotypes. Three among 11 polymorphic primers exhibited clear differentiation between heat-tolerant and heat-sensitive genotypes. The marker CEDG147 distinguished both heat-tolerant and heat-sensitive accessions (**Figure 4**). It was amplified at 300 bp in the tolerant genotype EC 398889 and at 285 bp in the sensitive genotype LGG 460. Similarly, another marker, CEDG 247, also distinguished both the genotypes at 161 bp and 168 bp, respectively (**Figure 4**). Furthermore, CEDG 044, distinguish between tolerant and sensitive genotypes at 192 bp in EC 398889 and at 162 bp, in LGG 460 (**Figure 4**).

**Figure 5:**
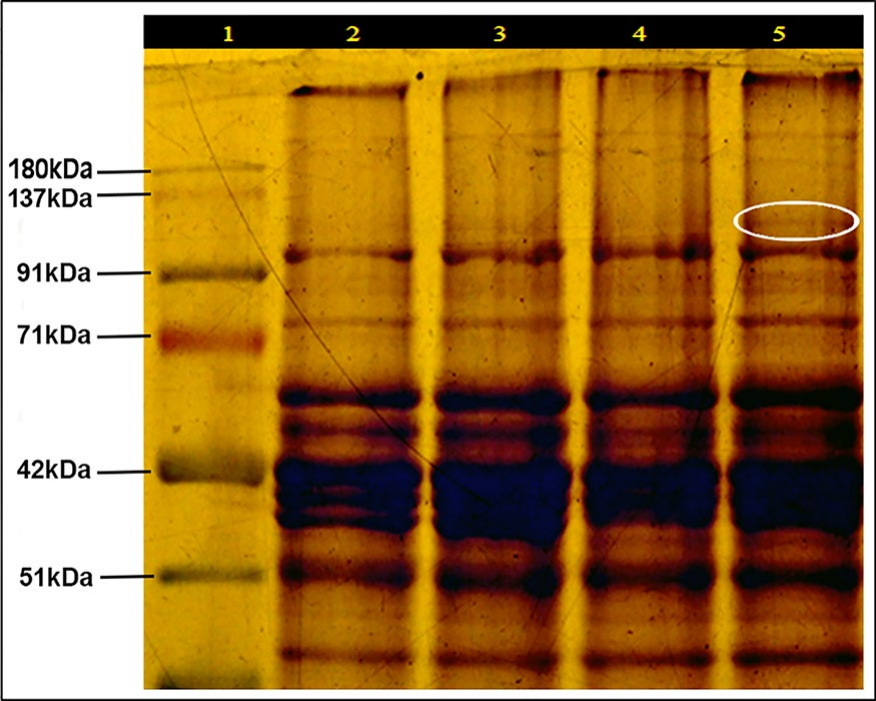
SDS-PAGE of protein profile (leaf) of heat sensitive (LGG 460) and tolerant (EC 398889) genotypes preadapted to normal (25/18 ^0^C) and high temperature (43/35^0^ C) conditions. Lanes of SDS-PAGE represented Protein markers (1); while (2 and 3) for LGG 460 adapted to low (25/18^0^C) and high (43/35^0^C) temperature (3), respectively; The lanes (4 and 5) for EC 398889 adapted to low (25/18 ^0^C) and high (43/35^0^C) temperature, respectively. Additional protein band between 91–137 kDa appeared in EC 398889 adapted to high temperature regime (as shown in circle).

**Figure 6:**
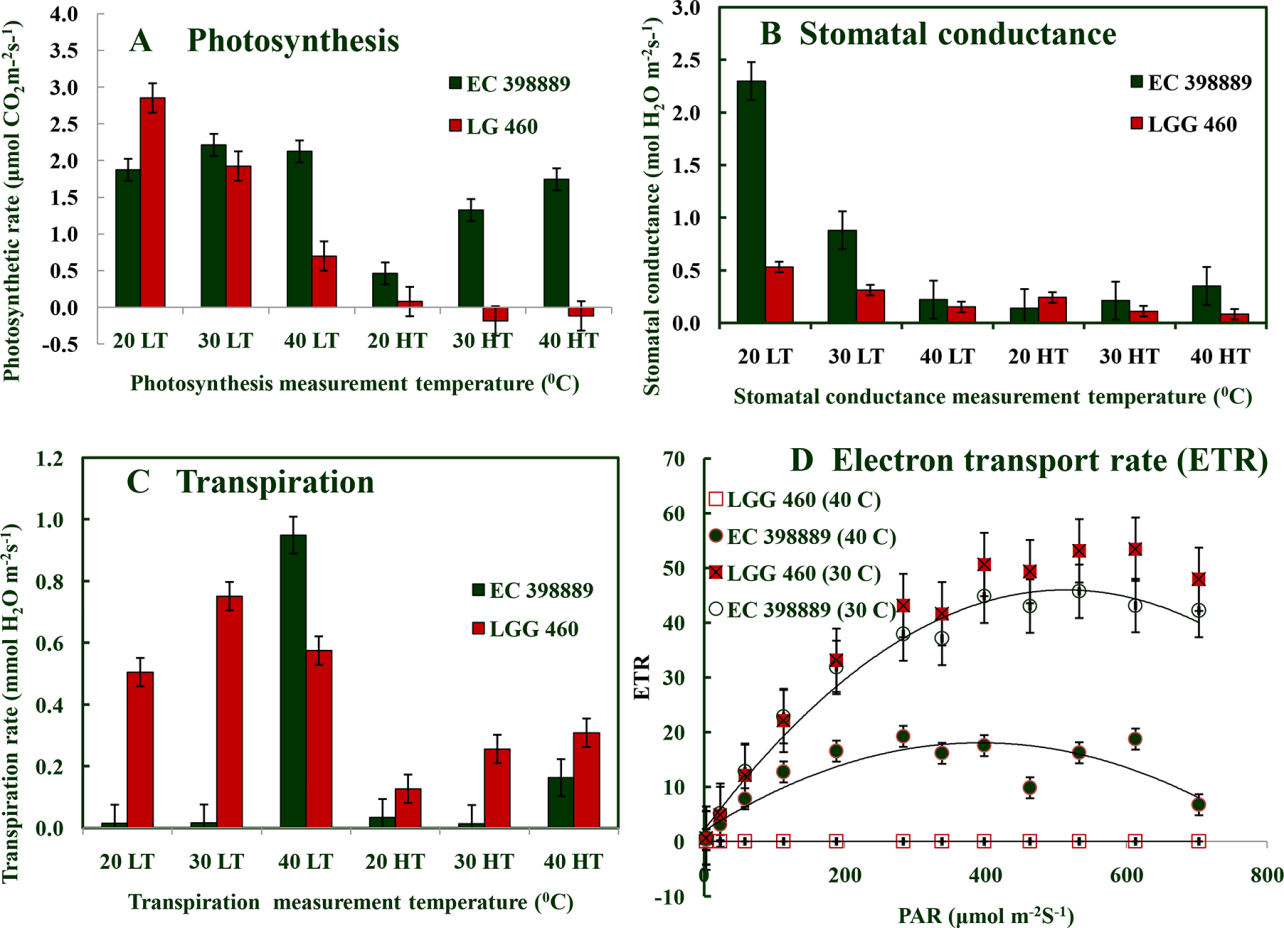
Response of net photosynthetic rate **(A)**, stomatal conductance **(B)**, and transpiration rate **(C)** to increasing temperatures (20^0^C, 30^0^C, and 40^0^C) in preadapted plants to low temperature (LT) or high temperature (HT) conditions in heat tolerant (EC 398889) and heat sensitive (LGG 460) genotypes. **(D)** represents light response of photosynthetic electron transport rate, ETR 30^0^C and 40^0^C in heat tolerant (EC 398889) and heat sensitive (LGG 460) genotypes. **(A**–**C)** Each value represents mean of three replications with standard error of mean (SEm) shown by error bar. Analysis of variance test using two factors factorial design (Genotype,G and Temperature, T) showed significant interaction effects (GxT) at P ≤ 0.01 on photosynthesis, stomatal conductance and transpiration with CD values 0.76**, 0.32**, and 0.15**, respectively, for treatment mean comparison. While **(D)**, three factors such as Genotype,G (2), temperature,T (2), and irradiance levels, L (13) were taken into account to test the significance level of interaction among these factors (GxTxL) which was shown by CD value 2.1** (P ≤ 0.01) for treatment mean comparison.

SDS-PAGE of leaf protein extracted from heat-sensitive (LGG 460) and heat-tolerant (EC 398889) greengram genotypes grown under controlled environment chamber (25°C/18°C) and (43°C/35°C) with 14-h photoperiod was conducted to identify the differences in protein profiles of these two contrasting genotypes. An additional protein band between 91–137 kDa was detected in the genotype EC 398889 under heat-shock (shown in circle) (**Figure 5**). Light-saturated rates of photosynthesis (P_max_) in LT-adapted plants of LGG 460 showed a progressive reduction in photosynthesis from 20°C to 40°C, and P_max_ drastically declined in HT-grown plants of LGG 460 (**Figure 6A**). By contrast, the LT-grown EC 398889 showed no reduction in the P_max_ within the range of test temperature 20°C–40°C, while P_max_ progressively increased with increasing test temperatures from 20°C to 40°C in HT-grown plants of EC 398889 (**Figure 6A**). Stomatal conductance in LGG 460 in both LT- and HT-grown plants decreased with a progressive increase in the test temperatures from 20°C to 40°C (**Figure 6**). However, despite the reduction in stomatal conductance, the P_max_ in LT- and HT-grown plants of EC 398889 did not proportionately decrease. However, photosynthesis increased with a progressive increase in the test temperature from 20°C to 40°C (**Figures 6A, B**). The transpiration rate in HT-grown LGG 460 increased along with the increase in the test temperatures (**Figure 6C**); however, negative photosynthesis (**Figure 6A**) and high transpiration (**Figure 6C**) in HT-grown LGG 460 appeared detrimental for the genotype in terms of negative carbon gain and more water loss. By contrast, in spite of substantial reduction in the stomatal conductance in HT-grown EC 398889 (**Figure 6B**) and relatively higher transpiration rate (**Figure 6C**), the P_max_ remained considerably higher, which indicated an enhanced capacity for photosynthesis in EC 398889 at HTs. Light response of photosynthetic electron transport rate (ETR) in EC 398889 and LGG 460 is shown in **Figure 6D**. The nonstomatal components of photosynthesis were assessed using quantum yield (Fv/Fm) and a final conversion into ETR for targeting the possible sites of action at the chloroplast level as a consequence of HT. Interactive effects of genotype × temperature × irradiance levels was significant and LGG 460 leaves treated at HT 40°C showed complete inhibition of photosynthetic ETR at all irradiance levels, while partial inhibition was noted in the heat-treated leaves of EC 398889 (**Figure 6D**).

**Figure 7:**
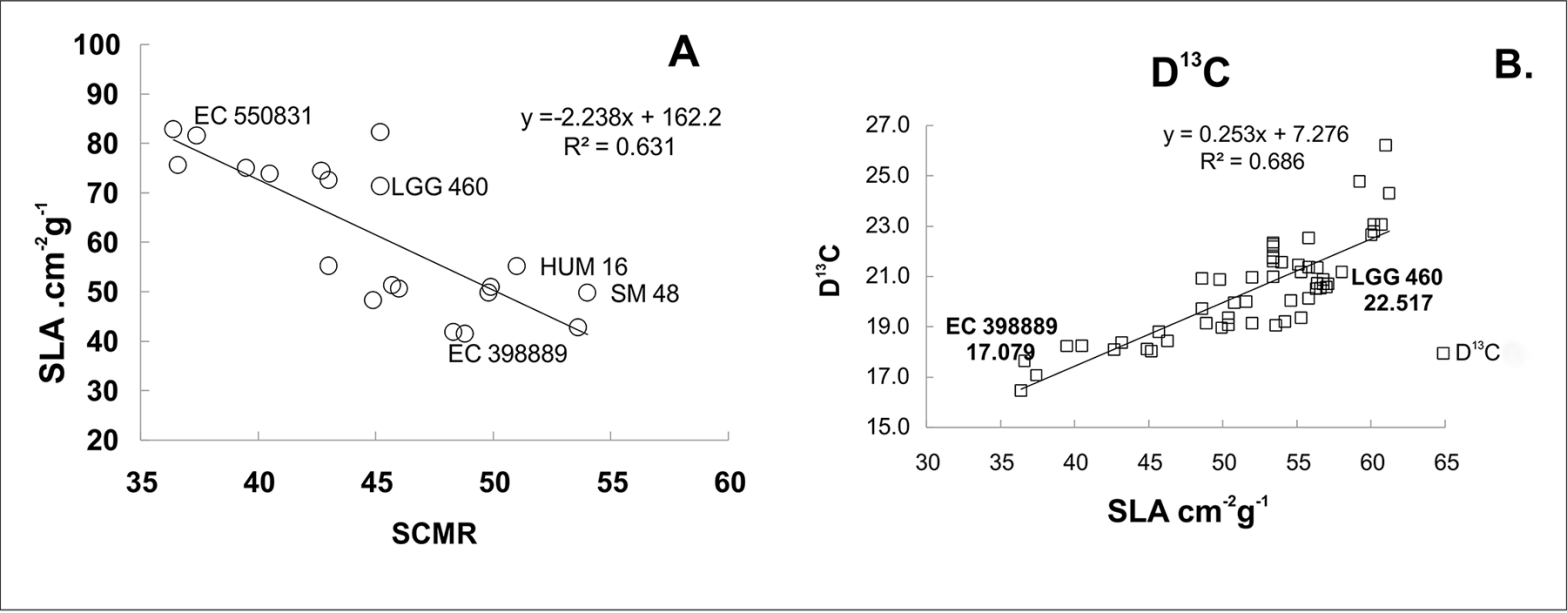
Relationship among specific leaf area (SLA), SPAD chlorophyll meter reading (SCMR), and carbon discrimination (D^13^C) in selected greengram genotypes. The heat tolerant genotype (EC 398889) showed lower, SLA **(A)** and D^13^C **(B)** values indicating higher photosynthate partitioning and water-use efficiency as compared to heat sensitive genotype (LGG 460) **(A**, **B)**. Each value represents mean of five replications.

**Figure 8:**
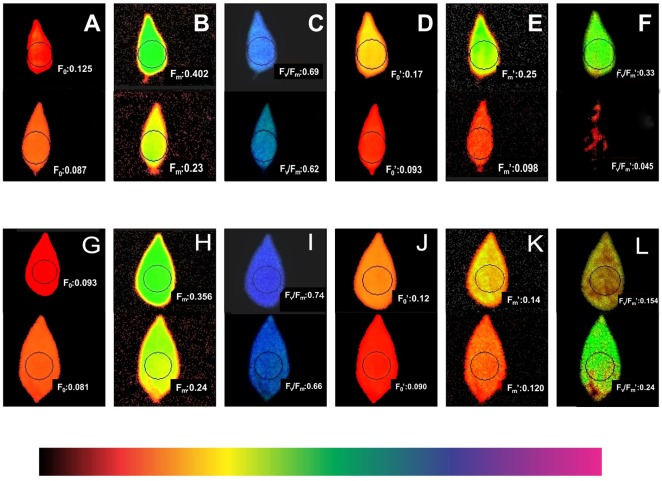
Changes in chlorophyll fluorescence images (F_0_, minimal; F_m_, maximal, and F_v_/F_m_, ratio of variable to maximal fluorescence or quantum yield) at two temperatures (30^0^C normal and 43^0^C high temperature) in heat tolerant (EC 398889) and heat sensitive (LGG 460) genotypes. (**A**–**C**; top view): Fluorescence images at 30^0^C in dark-adapted leaves (LGG 460). (**D**–**F**; top view): Fluorescence images at 30^0^C in light-adapted leaves (LGG 460). (**A**–**C**; bottom view): Fluorescence images at 43^0^C in dark-adapted leaves (LGG 460). (**D**–**F**; bottom view): Fluorescence images 43^0^C in light-adapted leaves (LGG 460). (**G**–**I**; top view): Fluorescence images at 30^0^C in dark-adapted leaves (EC 398889). (**J**–**L**; top view): Fluorescence images at 30^0^C in light-adapted leaves (EC 398889). (**G**–**I**; bottom view): Fluorescence images at 43^0^C in dark-adapted leaves (EC 398889). (**J**–**L**; bottom view): Fluorescence images at 43^0^C in light-adapted leaves (EC 398889).

Higher light harvesting efficiency was observed in the heat-tolerant genotype EC 398889 than in LGG 460 in the field-grown crop. Fresh leaf area per unit dry matter weight, which is known as SLA, was considerably lower in EC 398889 than in LGG 460 along with an increase in the SCMR (SPAD chlorophyll meter reading) value (**Figure 7**) at the podding stage, which indicated a higher light harvesting capacity and higher production of dry matter per unit leaf area in EC 398889 than in the genotype LGG 460. The SLA was negatively correlated with SCMR (**Figure 7A**), while SLA was observed to share a positive correlation with delta carbon (**Figure 7B**), which indicated that a lower SLA was associated with lower delta carbon (carbon isotope discrimination) and further suggested that higher water-use efficiency (WUE) was seen in heat-tolerant genotype EC 398889 than in the genotype LGG 460. Lower SLA with lower delta carbon values proved to be unique physiological attributes contributing tolerance to the genotype EC 398889 to adapt effectively by escaping terminal heat stress (**Figure 7**). Fluorescence images of dark- and light-adapted leaves was performed in both the genotypes treated at 30°C and 43°C for 1 h and images were captured for investigating the changes in fluorescence parameters, such as minimal fluorescence (F_0_), maximal fluorescence (Fm), and quantum yield denoted by the ratio of the variable fluorescence (Fv) to maximal, Fm fluorescence (Fv/Fm) affected by temperature change (**Figure 8**). Fluorescence images of heat-shocked leaves (43°C) were compared with those of normal temperature (30°C) treated leaves as checks. The numerical values of fluorescence parameters along with changes in the colour code have been interpreted as the degree of damage to the photosynthetic system caused by HT. More damaging effect was observed in light-adapted leaves treated at HT 43°C as quantum yield (Fv/Fm) declined from 0.62 (**Figure 8I**) to 0.045 (**Figure 8L**) and this changes in the fluorescence parameters were also substantiated by changes in false colour code (color bar indicating high to low values from right to left). The reduction in the Fv/Fm in the heat-shocked leaves of LGG 460 was almost 100%, thus indicating that the magnitude of heat stress >40°C could be lethal or detrimental for this genotype LGG 460 to sustain photosynthesis (**Figure 8L**). By contrast, better heat adapted genotype EC 398889 showed reduction in the quantum yield (Fv/Fm) from 0.66 to 0.24 after the heat shock and the partial inhibition of quantum yield (approximately 63%) depicted by Fv/Fm images (**Figure 8L**). The results revealed that threshold temperature at which photosynthetic system irreversibly changed in greengram could 43°C, however, genetic diversity for heat tolerance trait is evident in the present investigation.

**Figure 9:**
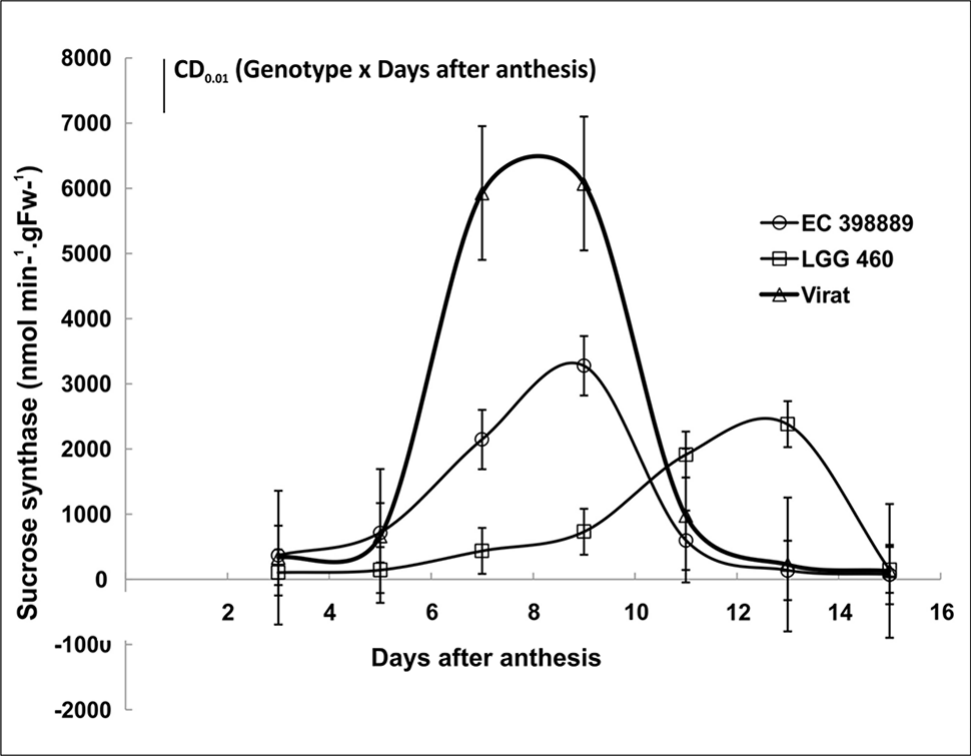
Sucrose synthase (SuSy) activity in developing grains of field-grown greengram (heat tolerant EC 398889 and sensitive LGG 460) genotypes at different pod development stages. Each value represents mean of three replications.Treatment means comparison (Genotype, G and days after anthesis, D) and significance levels of difference (CD) between genotype and days after anthesis activating SuSy was performed based upon the CD value 751.1** of interaction effects of Genotype x days (GxD) significant at P ≤ 0.01.

**Figure 10:**
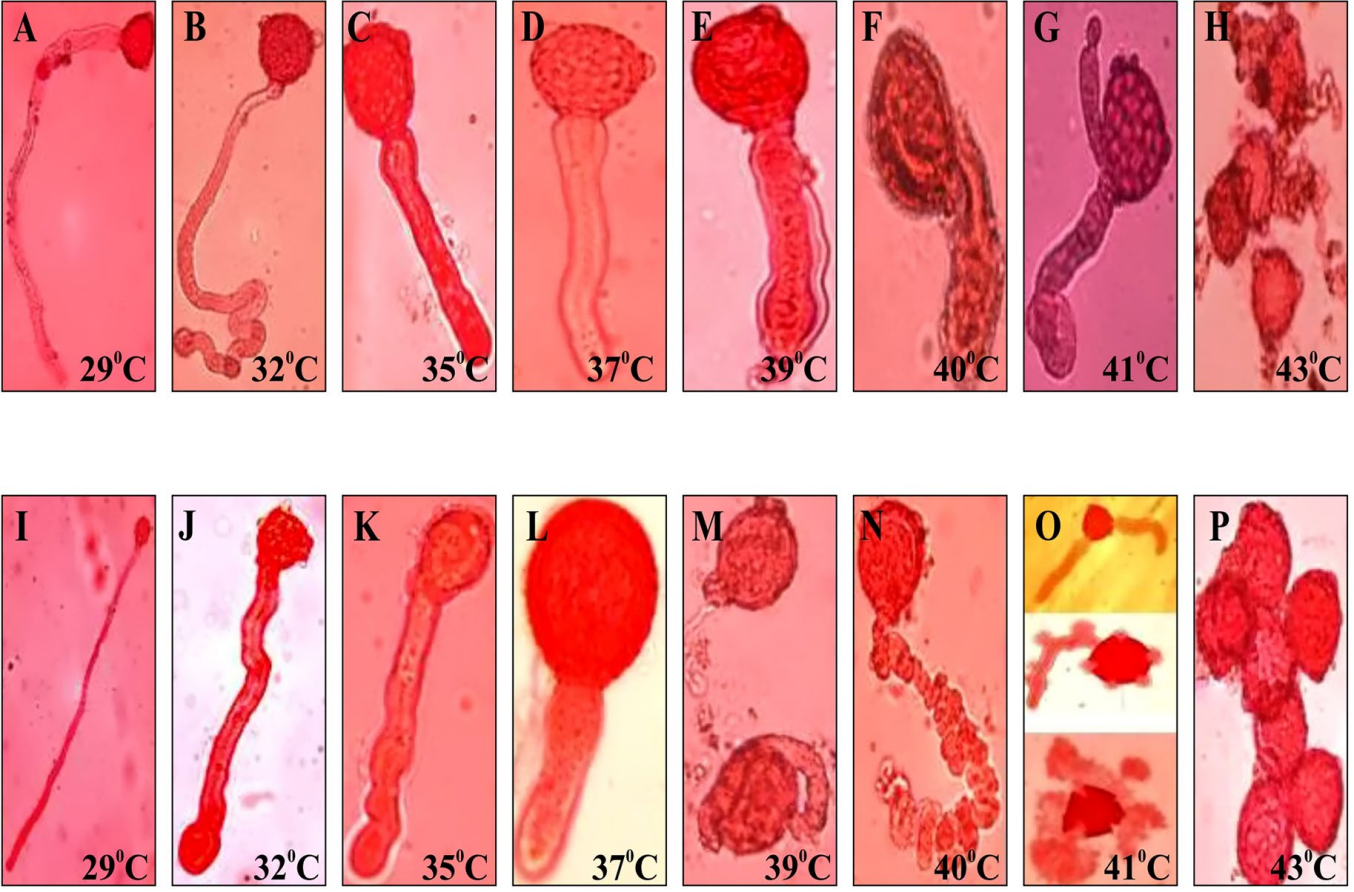
Pollen germination of heat tolerant (EC 398889) and sensitive (LGG 460) greengram genotypes at different temperatures. **(A,B,C,D,E,F,G,H)** represents pollen tube growth of heat tolerant (EC 398889) and **(I,J,K,L,M,N,O,P)** for heat sensitive (LGG 460) genotypes with progressive increase in the temperature from 29^0^C to 43^0^C.Abnormalities in pollen tube growth in heat sensitive genotype LGG 460 was noticed at much lower temperature starting from 39^0^C onwards than heat tolerant ones EC 398889 that had occurred at higher temperature 43^0^C.

Sink strength under stress is a crucial factor determining grain yield. To investigate the sink efficiency, one of the key enzyme sucrose synthase (SuSy) was targeted and activity was measured at different developmental stages in both the genotypes grown during summer. Significant differences in SuSy activity at different developmental stages were noticed. The SuSy activity in LGG 460 remained low after anthesis till Day 10 of anthesis and after that attained high activity. However, SuSy was extremely high even after 5^th^ day of anthesis in tolerant genotype EC 398889 attaining maximum activity on Day 8 or 9 and declined to an extremely low activity state when pods were near maturity (**Figure 9**). Thus, pod fill duration appeared to be regulated by time-dependant activation state of SuSy, and the two genotypes could be differentiated by the early or late upregulation of SuSy immediately after anthesis. The effect of HT on pollen germination was also investigated in contrasting greengram genotypes. With a progressive increase in the temperature beyond 35°C, the length of the pollen tubes decreased, diameter of tubes increased, and pollen sap became denser and more viscous, which results in poor mobility of pollen sap or slowdown of cytoplasmic streaming (**Figure 10**). Anthesis/fertilization might also have been affected by altered physiological changes that occur at HTs. Abnormalities such as coiling of pollen tubes, emergence of multiple tubes or bursting of pollen cell sap from multiple sites were observed in the insensitive line LGG 460 beyond 37°C (**Figure 10**). Most of physiological features in pollen germination that were affected by HT remained similar in both the genotypes. However, the genotype EC 398889, which is better adapted to HTs than LGG 460, showed normal growth and fertile pollens even at 43°C unlike LGG 460, which showed complete pollen sterility as seen in LGG 460 at 43°C (**Figure 10**).

## Discussion

The panel of greengram genotypes, constituting 116 diverse germplasms was classified into four broad clusters, which revealed wide genetic diversity of the greengram population under study,and genotype EC 398889 belonging to cluster 1 and LGG 460 belonging to cluster 3 were not linked with each other or distantly related in terms of the phenology and yield attributing traits (**Table 1**). The large variation in grain yield between the selected greengram experimental sites, Vamban and Kanpur in India, suggested the presence of ample genetic variability for various yield and yield-contributing traits (**Table 1**). The mean grain yield of 116 genotypes at Vamban was almost half of the mean grain yield at Kanpur although Vamban has better environmental conditions than does Kanpur with respect to temperature (38°C/21°C maximum/minimum) and humidity (approximately 70%) prevailed during crop growth period and no other abiotic factors could have affected the yield except for the day length, which was shorter by 1to 2 h in Vamban in comparison to Kanpur. Questions of whether the temperature regime during the crop season or day length at Vamban was not favorable and both the factors might have played a crucial role in determining the grain yield are likely to arise. Some of the questions are as follows: What caused Kanpur to consistently record higher yields than Vamban? Should different breeding strategies be adopted to develop greengram varieties? What traits are crucial for improving yield as well as enhancing the yield stability across environments? The duration of specific stages of growth appeared to have direct relationship with temperature because early growth stages of the crop at Vamban experienced higher temperature with max >35°C and min >25°C coupled with a shorter photoperiod of 11:30 h than Kanpur. Consequently, the combined effect on the crop was that the attainment of reproductive stage occurred considerably earlier because flower initiation occurred 3 to 10 days earlier than in Kanpur (**Table 2**). The results corroborated with earlier reports indicating that high mean temperature hasten flowering and, a low mean temperatures delay flowering at all photoperiods. Flowering is often progressively delayed in greengram when the photoperiod is extended (Pratap et al., 2013; Pratap et al., 2014). The reports suggested that long day length at Kanpur (12–14 h) delayed flowering, while short day length in Vamban (11–13 h) induced early flowering and maturity. Although greengram possess an indeterminate growth habit characterized by alternate flushes of flowers followed by vegetative growth, early flowering tends to shorten the crop cycle and favor early maturity with a substantial yield penalty. Evidently, Kanpur had drier weather (40%–50% RH) and longer photoperiod, which contributed higher biomass or vegetative growth at the early stages and the maximum temperature reached beyond 40°C (**Figure 1**). These results suggested that crops grown at Kanpur were adequately supported by vegetative biomass that had accumulated before flowering, and this could be one of the major yield determinants as is evident by observing a high yield at Kanpur although terminal heat stress at Kanpur had been more severe than that at Vamban, exceeding 40°C. In the context of yield improvement in greengram under these two contrasting environments, two-pronged strategies should be developed because Vamban does not experience terminal heat stress during the reproductive phase of the crop. Consequently, productivity could be substantially enhanced by introducing photoinsensitive and thermoinsensitive varieties, thus allowing substantial biomass to support grain filling. By contrast, heat-tolerant varieties are necessary for higher latitudes like Kanpur, where recurrent heat episodes are a regular feature.

The controlled environment studies are crucial for determining the effect of a specific environmental factor on yield and yield-contributing traits by eliminating the effects of other factors. In the present study, the controlled greenhouse conditions allowed the crop to grow at HT regime (45°C/25°C) with 14 h day length. Based on the results of field trials over 3 years at two locations, 17 genotypes with high stable yield and 11 with a stable but low yield were selected and evaluated in greenhouses (**Table 2**). The EC 398889 demonstrated the highest yield out of 17 putatively identified genotypes as stable high yield while the lowest yield was recorded in LGG 460 (**Table 2**).

Membrane stability index, as well as chlorophyll content or greenness index, remained higher in EC 398889 as compared with LGG 460 (**Table 3**). Under stress conditions, a sustained function of cellular membranes is considered crucial for maintaining cellular processes such as photosynthesis and respiration (Blum, 1998). The integrity and function of cell membranes are sensitive to HT, as heat stress alters structures of membranes proteins leading to increased permeability of membranes as evident from the increased loss of electrolytes in the test leaf samples (**Table 3**). The increased solute leakage is closely associated with cell membrane thermo- stability (Ilık et al., 2018), and various attempts have been made to use this method as an indirect measure of heat tolerance in diverse plant species such as food legumes (Srinivasan et al., 1996), soybean (Scafaro et al., 2010), potato, cotton, and tomato (Rahman et al., 2004; Hu et al., 2010), wheat (Blum et al., 2001).

**Table 3:** Membrane stability of selected greengram genotypes with stable high and low yield across the locations and over 3 years of experimentation.

Genotype	EC at 40°C	EC at 100°C	Membrane stability (%)	SPAD
China mung 1	95.3	301.4	31.62	21.1
IPM 2-14	84.3	253.4	33.27	38.2
UPM 02-17	67.9	268.0	25.34	22.2
Ganga 8	30.2	88.3	34.20	45.5
TARAM 2	29.2	138.7	21.02	32.6
EC 520011	37.5	136.9	27.42	22.1
EC398889	44.7	221.0	20.23	33.1
ML1256	34.6	178.7	19.36	24.7
IPM 99-125	23.6	114.4	20.61	15.2
Sona yellow	25.5	127.2	20.09	36.6
IPM 02-3	33.2	91.3	36.36	29.6
IPM 02-1	44.5	135.5	32.84	30.2
HUM 16	24.3	111.6	21.77	25.7
PDM 262	50.2	232.7	21.57	31.5
BM 4	36.4	194.6	18.71	43.9
GM 9911	27.6	142.5	19.37	27.3
PDM 87	34.6	178.7	19.36	18.4
LGG460	24.3	160.8	15.09	22.7
MH 2-15	23.2	162.3	14.32	14.6
NSB 007	23.1	194.7	11.88	25.2
Pusa 672	45.8	372	12.31	24.6
KM-2241	29.8	282.6	10.54	22.9
Kopergaon	34.6	320	10.81	15.7
CO4	27.6	172.6	15.99	13.8
SML 191	17.6	153.7	11.45	17.7
Vamban 3	30.6	412.1	7.43	14.3
		**CD (1%)**	**1.56**	**2.27**

The genotype EC 398889 was characterised by high acquired thermo- tolerance (76.8%) as compared with LGG 460 (34.5%). This was inferred based on the ability of TTC reduction by seedlings adapted to 37°C and 52°C for 2h (**Figure 3**). In addition to this, the heat- tolerant genotype had a unique attribute to start accumulating chlorophyll in cotyledonary leaves followed by regeneration of new leaves from the seedlings after severe heat shock (52°C), gradually tended to revive to normal plant after series of heat episodes from 37°C to 52°C (**Figures 3B, C**), however readjustment of physiological processes toward normalization took a long time for recovery. Seedlings that turned green and generated new leaves were scored as survivors. Thus, TTC and chlorophyll accumulation tests were found to be appropriate for monitoring sensitivity of a genotype to high- temperature stress. By contrast, heat-sensitive genotype LGG 460 failed to revive after episodic heat stress and completely lost cell viability as TTC test was found negative (**Figure 3D**). Our findings are in accordance with earlier reports indicating strong association of higher membrane thermo stability and cell viability after heat stress treatment of seedlings and the technique has been widely used for assessment of HT tolerance (Gupta et al., 2010). The TTC reduction assay measures the level of mitochondrial respiration activity, which serves as an indicator of cell viability (Berridge et al., 2005). Variability was detected among the 56 genotypes for acquired thermotolerance ranging from 14.1% to 61.3%.

The development of candidate gene markers for crucial heat tolerance genes may allow for the development of new cultivars with increased abiotic stress tolerance using marker-assisted selection (Pratap et al., 2015; Pratap et al., 2017; Jespersen et al., 2017). Molecular profiling of greengram accessions was also done using 79 SSR markers of which 11 were polymorphic. Among the polymorphic primers, three markers showed a clear differentiation between heat-tolerant and heat-susceptible genotypes. These markers exhibited a large amount of genetic variability among different accessions. The marker CEDG 147 distinguished tolerant and susceptible group of accessions and amplified at 300 bp in the heat-tolerant genotype EC 398889 and at 285 bp in sensitive genotype LGG 460. Similarly, another marker CEDG 247 also distinguished heat-tolerant and heat-susceptible genotypes at 161 and 168 bp, respectively. Likewise, marker CEDG 044 distinguished between tolerant and sensitive genotype at 192 and 162 bp, respectively (**Figure 4**).

SDS-PAGE of leaf protein extracted from LGG 460 and EC 398889 grown under controlled environment chamber (25°C/18°C) and (43°C/35°C) with 14 h photoperiods performed. An additional protein band between 91-137 kDa was detected in the genotype EC 398889 (shown in the circle), whereas this band was absent in LGG 460 (**Figure 5**). However, because this additional band was not thoroughly characterized, it could be inferred that the expression of this protein might have some role as a protective mechanism. This protein band was extremely close to the size of approximately 100–105 kDa. The result also showed the expression of one heat shock protein (HSP) of molecular size of 101 KDa (**Figure 5**) in the heat-tolerant greengram genotype EC 398889, which was consistent with earlier studies (Yoshida et al., 2011). Expression of various HSPs is an adaptive strategy in heat tolerance. Some HSFs (Hsp101, HSA32, HSFA1, and HSFA3) are critical for thermotolerance and play a crucial role in stress signal transduction, protecting and repairing damaged proteins and membranes, protecting photosynthesis as well as regulating a cellular redox state (Wang et al., 2004; Chi et al., 2019). The expression of various HSPs is known to be an adaptive strategy in heat tolerance. Hsp101 has been considered to be a molecular chaperone that impart heat tolerance to plants (Schlesing et al., 1982; Suk and Elizabeth, 2001), furthermore, it has special significance in maintaining proper conformation of proteins and facilitates the survival of organisms in high‐temperature stress. HSPs are induced by heat and strongly linked to heat tolerance (Yıldız and Terzioğlu, 2006). Different classes of HSPs play different roles in protection from stress; however, most HSPs serve as chaperones.

The genotype LGG 460 could not adapt at high thermal regimes (maximum/minimum) 40°C/30°C in a controlled environment because photosynthesis (P_max_) was inhibited completely compared with LT-grown plants (25°C/18°C) (**Figure 6A**). The P_max_ was more adversely affected (**Figure 6A**) than stomatal conductance (**Figure 6B**) and transpiration (**Figure 6C**) in the HT-grown plants indicating involvement of nonstomatal components that were likely to be the factors responsible for inhibiting photosynthesis. By contrast, the genotype EC 398889 was not only adapted well under high thermal regime 40°C/30°C, instead P_max_ progressively increased when photosynthesis measured from lower (20°C) to higher (40°C) test temperatures (**Figure 6A**), and the temperature response of P_max_ was proportionate to relative changes in temperature response of stomatal conductance (**Figure 6B**) and transpiration (**Figure 6C**). Higher photosynthesis with low stomatal conductance and transpiration rate enabled more carbon gain over water loss; hence, WUE increased in heat-tolerant genotypes when subjected to stress (**Figure 6**). By contrast, sensitive genotype LGG 460 confronted with different situations, such as reduction of photosynthesis at HT, which was associated with an increase in the stomatal conductance and transpiration. Hence, no carbon gain per unit loss of water occurs, which suggested that the plants encountered multiple stresses, such as HT, light intensity, and drought (**Figure 8**). Photosynthesis is sensitive to HT (Kim and Portis, 2005) and the ability to sustain leaf gas exchange under heat stress is directly correlated with heat tolerance (Bita and Gerats, 2013). The reduction of active Rubisco and Rubisco activase could be responsible for the inhibition of photosynthesis (Maestri et al., 2002; Morales et al., 2003; Salvucci and Crafts-Brandner, 2004; Ristic et al., 2009), the carbon fixation is affected by limitation of Rubisco. The stroma and thylakoid membrane system are the most sensitive and primary target sites of heat injury (Maestri et al., 2002; Morales et al., 2003; Wise et al., 2004). Photosynthesis is the most thermosensitive plant function (Kim and Portis, 2005); hence, supraoptimal temperatures adversely affect photosynthesis. Photosynthesis can occur optimally at wide temperatures in the range 15°C–35°C, but it is adversely affected at temperatures exceeding 40°C. Chloroplast stroma and thylakoid membranes are damaged by HTs (Wang et al., 2010). Photosystem (PS)II in the light reaction (Heckathorn et al., 2002) and Rubisco (ribulose1, 5-bisphosphate carboxylase/oxygenase) activase in the Calvin cycle (Crafts-Brandner and Salvucci, 2000) are both thermo-labile. Heat stress thus impairs the electron transport chain and affects the activation and activity of the enzyme Rubisco (Ahmad et al., 2010). Although PSI and PSII are both adversely affected by HTs, but PSII is more sensitive to heat stress than is PSI (Moustaka et al., 2018).

The first distinct change in both structure and function of photosystem II (PSII) reported to be occurred at 40°C –50°C in barley (Lípová et al., 2010). The first temperature induced transient changes had been shown at 42°C to 48°C with a disruption of the PSII donor side and corresponding loss of oxygen evolution (Cramer et al., 1981) followed by changes in thylakoid membranes at about 60°C and loss of electron transport through PSII (Smith et al., 1989) representing a denaturation of the PSII reaction centers. At about 75°C, a denaturation of light-harvesting complex of PSII(LHCII) has been observed (Smith et al., 1989).

In the present study, inhibition of photosynthesis at HT was assessed through gaseous exchange as well as chlorophyll fluorescence imaging, which indicate the effects of stress on PS II photosynthetic membrane system and ETR. The light response of ETR at two pretreatment temperatures, namely, 30°C and 40°C, is shown in **Figure 6D**. The ETR in HT pretreated leaves (40°C) of EC 398889 never declined to zero with progressive increase in irradiance levels (**Figure 6D**). However, heat-sensitive genotype LGG 460 showed complete reduction of ETR at all levels of irradiance when pretreated at 40°C. Reduced electron transport and damaged photosystems caused by high temperature have been reported in poplar by Song et al. (2014).

The genotype EC 398889 had low SLA (leaf area g^−1^ leaf weight). Furthermore, it had a high SCMR or greenness index, which suggested higher chlorophyll levels within a smaller leaf surface area, which enabled the plant to absorb more solar radiation per unit area of leaf in comparison with genotype LGG 460 (**Figure 7**). More chlorophyll per unit of leaf area in EC 398889 was likely to enhance photosynthesis than in the genotypes having higher SLA and low SCMR, such as LGG 460 (**Figure 7**). SLA was also positively correlated with delta carbon indicating that lower values of delta carbon are associated with low SLA values. High radiation-use efficiency and high WUE are attributed to low SLA coupled with low delta carbon values, as exhibited by EC 398889 (**Figure 7**). SLA has been reported to be associated with variation in photosynthetic capacity and chlorophyll density (Nageswara Rao et al., 2001; Kalariya et al., 2015). SCMR contributes to high photosynthesis and ultimately to increased yield (Arunyanark et al., 2009). Koolachart et al. (2013) reported that SLA indicates high chlorophyll content in leaves that contribute to high photosynthesis and yield.

The fluorescence parameters (F_0_, Fm, and Fv/Fm) were altered because of heat treatment at 43°C in dark and light-adapted leaves in the heat-tolerant and heat-sensitive genotypes. The modification of chlorophyll florescence in response to heat stress has been reported in numerous crops, and heat tolerance of plant species can be quantified by measuring chlorophyll florescence (Willits and Peet, 2001). Complete inhibition of quantum yield (Fv/Fm) of photosystem II was observed in light-adapted leaves pretreated at 43°C in genotype LGG 460 (**Figure 8L**), however, light-adapted leaves of EC 398889 that were subjected to the same treatment showed reduction in quantum yield (Fv/Fm) by approximately 64% (**Figure 8L**) compared with dark-adapted leaves (**Figure 8I**) immediately after heat shock. This finding suggested that light is an additional stress, and when leaves are exposed to heat shock, it becomes more detrimental to photosynthesis. The relative assessment of fluorescence images, particularly for quantum yield (Fv/Fm) after heat treatment, revealed that light-adapted leaves of the heat-tolerant greengram genotype EC 398889 exhibited higher quantum yield than the heat-sensitive genotype, LGG 460, as evidenced by fluorescence images for Fv/Fm. The photosynthetic system partially or completely collapsed in LGG 460 because no quantum yield (Fv/Fm) images were obtained with light-adapted leaves (**Figure 8L**). The fluorescence images combined with the light curve of ETR strongly suggested differential sensitivity of photosynthesis in the two contrasting genotypes (**Figure 8**).

The images of effective PS II quantum yield (YII) captured under high temperature and irradiance level were able to distinguish heat tolerant and susceptible genotypes. Similarly, the light response of electron transport rate (ETR) was also able to distinguish the genotypes based on their sensitivity to heat stress. Overall, this investigation indicates the suitability of chlorophyll fluorescence imaging system technique for precise phenotyping of greengram based on their sensitivity to heat stress. The findings are in accordance with earlier reports in rice (Pradhan et al., 2019) and wheat (Brestic et al., 2012).

Differential degree of membrane thermostability may distinguish the genotypes towards different sensitivity to heat stress. Chen et al. (2018) reported that chloroplast-targeted AtFtsH11 protease plays critical roles for maintaining the thermostability and structural integrity of photosystems under high temperatures. Therefore, the photosynthetic efficiency may be modified under heat stress by improving FtsH11 protease in photosystems, hence, to improve plant productivity. Sucrose synthesis in developing grains plays a crucial role in sink development and also determines the sink strength in several crops. It also acts as a signal molecule for promoting the conversion of transported sugar into starch. In the present study, sucrose synthase activity at different developmental stages differed among the test genotypes (**Figure 9**). The activity of sucrose synthase in developing grains of LGG 460 remained low and followed a long lag phase till day 10 of pod setting, while the genotype EC 398889 and a variety named “Virat,” which was derived using this genotype as the male parent showed a sharp increase in the activity of sucrose synthase after day 5 of pod setting with a concomitant increase in the sucrose content in developing grains. The early activation of sucrose synthase in the test genotypes EC 398889 appeared to be responsible for rapid grain filling and pod development and is likely to be associated with early pod maturity. The availability of photosynthates and sucrose, the transportable sugar, could also be responsible for long lag phase kinetics of sucrose synthase activity in LGG 460 because decrease in photosynthesis also limits sucrose transport to the sink, which might have influenced sink development at HT. Thus, sink development is inhibited in heat-sensitive genotypes. Hence, the first step in the conversion of sucrose to starch is likely to be primarily catalysed by sucrose synthase. These results also suggested that sucrose synthase activity could be considered a marker for sink strength. The enzymes responsible for metabolising sucrose may regulate sucrose import into the sink. High activities of sucrose-metabolising enzymes could increase the sucrose gradient; consequently, large amounts of sugar are imported for metabolism and storage. Wang et al. (1993) emphasised the importance of sucrose synthase rather than acid invertase as the dominant enzyme in metabolising imported sucrose in a growing sink. Sucrose synthase is responsible for the breakdown of sucrose, thus providing intermediates for the synthesis of starch and other polysaccharides. Reduced sucrose metabolism under high temperatures has been attributed to the changes in sucrose synthase and invertase (Dai et al., 2015).

Many legumes and cereals exhibit a high sensitivity to heat stress during flowering. One of the major yield determinants in greengram is pollen fertility and flower shedding at a HT. The length and size of the pollen tube and density of the pollen sap appeared to be altered by a progressive increase in the temperature beyond 37°C (**Figure 10**). The effect of HT on pollen germination was characterized by transformation of pollen sap into a dense and viscous fluid that probably hinders the smooth movement of male gametes. In addition to a reduction in the length of pollen tubes, no other pollen abnormalities were observed in the heat-tolerant genotype EC 398889 up to 40°C (**Figure 10**). By contrast, multiple abnormalities were detected in pollen tubes of the heat-sensitive genotype LGG 460, where the emergence of multiple tubes, and their bursting and coiling were observed; eventually, the pollen failed to germinate at temperatures exceeding 40°C (**Figure 10**). Earlier reports on rice have also indicated that an increase in temperature could limit yield by affecting pollen germination and grain formation (Endo et al., 2009; Wassmann et al., 2009; Chakrabarti et al., 2010). The male gametophyte is particularly sensitive to HTs at all stages of development, while the pistil and the female gametophyte are considered to be more tolerant (Hedhly, 2011). The sensitivity of pollen grains to temperature damage could be considered a crucial parameter for predicting rice yield in warmer climates. In legumes, heat stress during post-anthesis results in poor pollen germination on the stigma and reduced pollen tube growth in the style (Talwar et al., 1999). Under HT (30°C), flower sterility has been correlated with diminished anther dehiscence, poor shedding of pollen, poor germination of pollen grains on the stigma, reduced elongation of pollen tubes, and reduced *in vivo* pollen germination (Fahad et al., 2015; Fahad et al., 2016). High temperature decreases pollen viability and leads to sterile pollens and decrease of pod set and yield (Hasanuzzaman et al., 2013), as pollens are most sensitive to high temperature, the crop yield is affected when temperature rises during pollen development (Ploeg Van der and Heuvelink, 2005). The observed reduction in photosynthesis, in the present study, in heat sensitive genotype LGG 460 under high temperature might restrict accumulation of desired level of essential carbohydrates such as sucrose, hexoses and starch in the developing pollens, as a result pollen germination or fertility is adversely affected. The role of sugars and invertase/sucrose synthase activity in anther development and pollen germination has been reported in several crops (Goetz et al., 2001; Castro and Clement, 2007; Pressman et al., 2012; García et al., 2013; Le Roy et al., 2013; Singh and Knox, 2013). In the present study, Photosensitive character was eliminated by the series of field and controlled environment trials and eventually putative photoinsensitive lines were selected for evaluating their thermotolerance. These contrasting greengram lines, namely, EC 398889 and LGG 460, proved to be extremely valuable germplasm resources for crossing programmes.

## Conclusion

The changes in photothermoperiods across locations and under high-temperature stress during the reproductive stage have been considered to be major yield destabilizing factors in greengram. Three consecutive years of field experiments using a set of 116 genotypes at two locations, namely, Vamban and Kanpur in India, differing in day length and thermal regimes led to the identification of a few promising genotypes with stable high or low yield, depending on their relative insensitivity towards photothermoperiods based on two location data. Thermotolerance of selected genotypes was assessed under HT conditions simulated in a naturally-lit greenhouse. After vigorous testing, two contrasting genotypes were found to differ primarily in pod setting and grain yield at HT. The genotypes EC 398889 and LGG 460 exhibited the highest and lowest yield, respectively, based on their heat tolerance in addition to photo insensitivity exhibited by them based on multilocation trials. Heat tolerance and underlying mechanisms have been deciphered in these genotypes involving cellular thermal stability, ATT, chlorophyll fluorescence, pollen germination and photosynthesis, WUE, pollen fertility, and sink capacity. The results showed that source (leaf) efficiency could be enhanced by increasing the amount of chlorophyll per unit leaf, which means reducing SLA, which will improve WUE and minimise stomatal conductance and transpiratory water loss. Threshold temperature for tolerance for photosynthesis in greengram has been detected to the limit of 43°C based on photosynthetic ETR and other fluorescence parameters. Beyond 43°C, often irreversible changes is occurred in photosynthetic system. Faster activation of sucrose synthase in developing grains immediately postanthesis supported rapid grain filling and hastened pod maturity before the onset of HT. Therefore, modifications are necessary both at source and sink levels to improve productivity of greengram under changing climates.

## Data Availability Statement

The datasets for this article are not publicly available. The data is important, once published this data will be publicly available. Requests to access the datasets should be directed to psbsu59@gmail.com.

## Author Contributions

PB: Conception, designing, interpretation, drafting the manuscript, and conducting the experiments. AP, SG, and NS: Compilation of the results, editing and approval of final version. KS and RT: Conducted laboratory based experiments.

## Funding

The work was accomplished with financial support of the ICAR-National Innovations on Climate Resilient Agriculture (NICRA), Indian Council of Agricultural Research, New Delhi, India.

## Conflict of Interest

The research was conducted in the absence of any commercial or financial relationships that could be construed as a potential conflict of interest.
